# Zirconia-Toughened Alumina Ceramic Wear Particles Do Not Elicit Inflammatory Responses in Human Macrophages

**DOI:** 10.3390/ijms24076482

**Published:** 2023-03-30

**Authors:** Alessandro Alan Porporati, Yvonne Mödinger, Sarah Fischer, Sara Polajžer, Melanie Mettang, Ulrike Deisinger, Matejka Podlogar, Rihard Trebše, Nika Lovšin

**Affiliations:** 1 Medical Products Division, CeramTec GmbH, 73207 Plochingen, Germany; a.porporati@ceramtec.de (A.A.P.); y.moedinger@ceramtec.de (Y.M.); m.mettang@ceramtec.de (M.M.); u.deisinger@ceramtec.de (U.D.); 2 Department of Engineering and Architecture, University of Trieste, 34127 Trieste, Italy; 3 Medizintechnik, University of Stuttgart, 70174 Stuttgart, Germany; 4 Faculty of Pharmacy, University of Ljubljana, Aškerčeva 7, 1000 Ljubljana, Slovenia; 5 Jozef Stefan Institute, 1000 Ljubljana, Slovenia; mateja.podlogar@ijs.si; 6 Valdoltra Orthopaedic Hospital, 6280 Ankaran, Slovenia; rihard.trebse@ob-valdoltra.si; 7 Faculty of Medicine, University of Ljubljana, Vrazov trg 2, 1000 Ljubljana, Slovenia

**Keywords:** orthopaedic implant, alumina, zirconia-toughened alumina (ZTA), nanoparticles, macrophages, cell viability, inflammation, cytokine secretion

## Abstract

Ten percent of patients undergoing total hip arthroplasty (THA) require revision surgery. One of the reasons for THA are wear particles released from the implants that can activate the immune defense and cause osteolysis and failure of the joint implant. The discrepancies between reports on toxicity and immunogenicity of the implant materials led us to this study in which we compared toxicity and immunogenicity of well-defined nanoparticles from Al_2_O_3_, zirconia-toughened alumina (ZTA), and cobalt chrome (CoCr), a human THP-1 macrophage cell line, human PBMCs, and therefrom-derived primary macrophages. None of the tested materials decreased the viability of THP-1 macrophages nor human primary macrophages at the 24 h time point, indicating that at concentrations from 0.05 to 50 µm^3^/cell the tested materials are non-toxic. Forty-eight hours of treatment of THP-1 macrophages with 5 µm^3^/cell of CoCr and Al_2_O_3_ caused 8.3-fold and 4.6-fold increases in TNF-α excretion, respectively, which was not observed for ZTA. The comparison between THP-1 macrophages and human primary macrophages revealed that THP-1 macrophages show higher activation of cytokine expression in the presence of CoCr and Al_2_O_3_ particles than primary macrophages. Our results indicate that ZTA is a non-toxic implant material with no immunogenic effects in vitro.

## 1. Introduction

Ten percent of patients undergoing total hip arthroplasty (THA) require revision surgery and prosthesis replacement up to 10 years after the primary THA [1]. Revision THA is associated with a three to eight times higher mortality rate, poorer clinical outcome, prolonged recovery, and much higher costs than primary surgeries [2]. Half of the primary THA revisions are because of aseptic loosening [3] caused by stress and motion-induced bone to implant interface debonding but also by particle disease. Wear particles, particularly from metallic implants, can activate the host immune defense, which can lead to osteolysis and finally to joint implant failure [4,5]. Cobalt chrome (CoCr) is a metal alloy commonly used in dental and orthopaedic implants in which wear debris and released metal ions are suspected to be causative for severe periprosthetic tissue inflammation and necrosis [6,7,8]. Zirconia-toughened alumina (ZTA), on the other hand, is a ceramic composite material widely used as biocompatible implant material, which has a low potential for pathological inflammatory processes due to low wear rate and biologic inertness of the wear particles [9,10,11,12,13]. Importantly, it is believed that the local toxicity of metal ions creates local immune deficiency by creating a microenvironment of immune cell migration, possibly also facilitating bacteria growth at the implant site [14]. Indeed, the presence of any implanted medical device reduces the required bacteria concentration to induce an infection by 100,000 times, and different patient characteristics (e.g., age or pre-existing health conditions) can further promote an infection [15]. Of note, with the exception of approximately 10% of detected infections in THA revisions [3], it is suspected that 15% up to 76% of aseptic loosening diagnoses are actually undetected, chronic low-grade periprosthetic joint infections (PJI) that are misdiagnosed and mistreated, e.g., due to a lack of reliable diagnostic tests [16,17,18,19]. The costs of PJI treatment can be up to USD 0.5 million [20]. Given the above-mentioned implant-associated inflammatory processes, different prosthetic materials have been tested for toxicity and immunogenicity in numerous in vitro studies by measuring viability and activation of immune cells (peripheral blood mononuclear cells (PBMCs), macrophages, macrophagic cell lines) and pre-osteoblast cells [4,21,22]. Macrophages and osteoblasts can phagocyte alumina (Al_2_O_3_) nanoparticles, which causes activation of an inflammatory response and cytokine release [23,24]. Several studies have shown that Al_2_O_3_ particles can activate the immune response; however, there is controversy in the reports about their toxicity and the effect on human cell lines. When immunogenicity of Al_2_O_3_ particles was compared to titanium nanoparticles, only titanium particles caused a significant upregulation of receptor activator of NF-kB (*RANK*)-, tumour necrosis factor-alpha (*TNF-α*)-, and osteoprotegerin (*OPG*)-mRNA in the THP-1 human monocytic cell line [25]. Al_2_O_3_ particles did not cause a significant increase of common inflammatory markers, such as TNF-α, in THP-1 macrophages [25]. Al_2_O_3_ nanoparticles were shown to not interfere with the viability of THP-1 macrophages and fibroblast cells lines [26]. Radziun et al. reported that Al_2_O_3_ nanoparticles are capable of penetrating the membranes of the L929 mouse fibroblast cell line and the BJ human fibroblast cell line without a significant decrease in cellular viability [26]. A recent report, however, in which THP-1 macrophages were treated with Al_2_O_3_ demonstrated that Al_2_O_3_ nanoparticles induced the inflammasome in THP-1 macrophages via activation of the Toll-like receptor 4 (TLR4) pathway [23]. In that study, 24 h treatment with Al_2_O_3_ or zirconia (ZrO_2_) at the concentration of 50 µm^3^/cell caused upregulation of interleukin 8 (*IL-8*), CC-chemokine ligand 2 (*CCL2*), *CCL3,* and *CCL4* expression while not affecting cell viability [23]. Another study reported a four-fold increase in TNF-α in THP-1 macrophages in the presence of Al_2_O_3_ particles [27]. When PBMCs were cultured on ceramic surfaces, an increase of cytokines IL-6 and IL-1β secretion was reported for alumina-toughened zirconia (ATZ) but not for ZTA [28]. Several studies have also reported that CoCr particles activate the TLR4-mediated inflammation mechanism [28,29,30].

Given the controversy of previously published data, we hypothesized here that the toxicity of metallic oxide particles highly depends on the size composition of particles and varies between different cell types. Therefore, we compared the immunogenicity of Al_2_O_3_, ZTA, and CoCr particles by investigating THP-1 macrophages and primary human macrophages. In detail, viability, cytokine gene expression, and cytokine secretion of cells were investigated 24, 48, and 96 h after treatment with different concentrations (0.05, 0.5, 5, and 50 µm^3^/cell) of the test particles.

## 2. Results

### 2.1. Al_2_O_3_ and ZTA Particles Do Not Affect THP-1 Macrophage Viability

**Figure 1:**
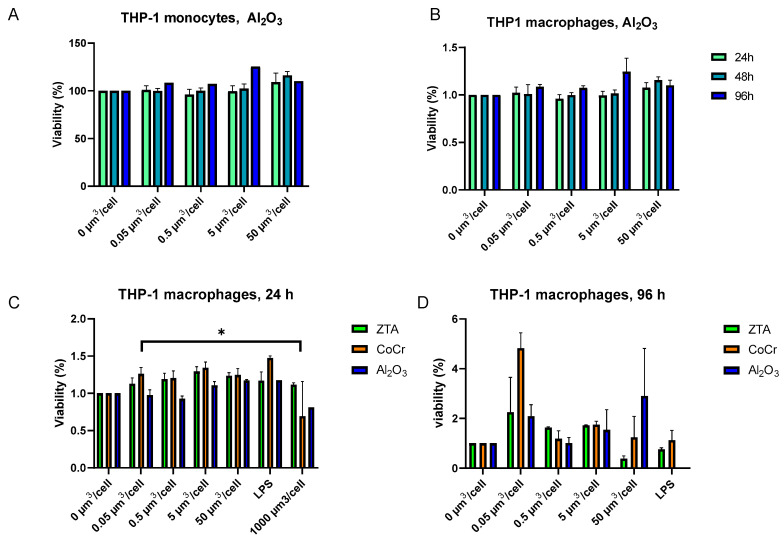
Al_2_O_3_ and ZTA particles do not affect THP-1 macrophage viability. Cell viability was measured by MTS assay. THP-1 monocytes (**A**) and THP-1 macrophages (**B**) were treated with Al_2_O_3_ particles for 24, 48, and 96 h (**A**). THP-1 macrophages were treated with ZTA, CoCr, or Al_2_O_3_ particles for 24 h (**C**) and 96 h (**D**). The results are represented as relative values in which untreated cells represent 100% viability. At least three biological repeats are depicted. The results are expressed as the mean ± SEM. *—*p* < 0.05.

We examined the viability of THP-1 macrophages after treatment with raw alumina (Al_2_O_3_) (CERALOX APA 05), ZTA, and CoCr particles. To examine whether experimental concentrations of particles (0.05, 0.5, 5, and 50 µm^3^/cell) have any impact on the viability of THP-1 monocytes and THP-1 macrophages, the viability of cells was measured at 24, 48, and 96 h post-treatment. Results show that Al_2_O_3_ particles neither affect the viability of THP-1 monocytes (Figure 1A) nor of THP-1 macrophages (Figure 1B) at any tested concentration or time point (Figure 1). When THP-1 macrophages were treated with ZTA or CoCr particles for 24 h, no difference in the viability was observed in comparison with untreated cells (Figure 1C; Al_2_O_3_ is additionally shown for comparison). Longer treatment for 96 h with equal concentration of the test particles did not significantly affect the viability of THP-1 macrophages either (Figure 1D). The addition of lipopolysaccharide (LPS), a positive stimulator of the immune response, also had no effect on cell viability (Figure 1D). However, when the concentration of CoCr particles was increased to 1000 µm^3^/cell, the viability of cells reduced by 0.54-fold (*p* = 0.019) in comparison with untreated cells. Of note, ZTA and Al_2_O_3_ particles did not show such an effect (Figure 1C). Altogether, our data indicate that Al_2_O_3_ and ZTA nanoparticles are not toxic for the human THP-1 cell line.

### 2.2. Treatment of THP-1 Macrophages with CoCr and Al_2_O_3_ Particles Caused an Increase in TNF-α Secretion

**Figure 2:**
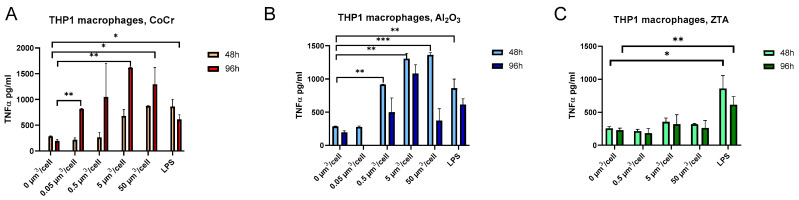
TNF-α secretion by THP-1 macrophages is activated by CoCr and Al_2_O_3_ particles but not by ZTA particles. THP-1 macrophages were treated with the test particles for 48 h or 96 h, and the concentration of secreted TNF-α was measured in the cell supernatants by ELISA. Treatment with CoCr particles (**A**). Treatment with ZTA particles (**B**). Treatment with Al_2_O_3_ particles (**C**). LPS was used as a positive control. The results are expressed as the mean ± SEM. *—*p* < 0.05; ** *p* ≤ 0.01; *** *p* ≤ 0.001.

Next, the immune response of THP-1 macrophages to the test particles was examined by measurement of TNF-α secretion into the cell supernatant by ELISA. THP-1 macrophages were treated with the particle concentrations of 0.05, 0.5, 5, and 50 µm^3^/cell for 48 h or 96 h. When cells were treated for 48 h with CoCr particles at a concentration of 50 µm^3^/cell, a three-fold (*p* = 0.02) increase in TNF-α level was observed in comparison with untreated cells, which was comparable to LPS treatment (positive control). Treatment for 96 h led to a TNF-α increase at even lower concentrations of CoCr nanoparticles (8.3-fold change (*p* = 0.0003) at 5 µm^3^/cell and 4.4-fold change (*p* = 0.0018) at 0.05 µm^3^/cell, respectively) in comparison with untreated cells (Figure 2A). Treatment of cells with 5 µm^3^/cell and 50 µm^3^/cell Al_2_O_3_ particles for 48 h caused 4.6-fold and 3-fold (*p* = 0.0003) increases in TNF-α level, respectively (Figure 2B). Longer treatments (96 h) of THP-1 macrophages with Al_2_O_3_ caused an increase in excreted TNF-α already at the concentration of 0.5 µm^3^/cell (5.5-fold change, *p* = 0.0228). On the other hand, treatment of THP-1 macrophages with ZTA particles did not cause an increase in TNF-α, neither at 48 h nor at 96 h, while the addition of LPS caused a significant increase in TNF-α after 48 h (3.4-fold (*p* = 0.0123)) and 96 h (2.7-fold, *p* = 0.001) treatment (Figure 2C). 

### 2.3. Al_2_O_3_, ZTA, and CoCr Particles Do Not Impact Cell Viability of Human Primary Macrophages and PBMCs

**Figure 3:**
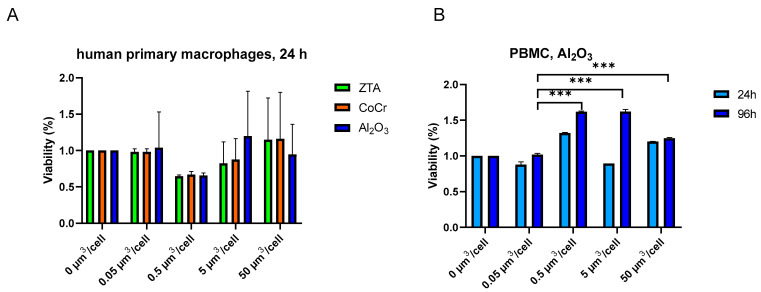
Viability of human primary macrophages is not impacted by ZTA or CoCr particles. Human primary macrophages were treated with Al_2_O_3_, ZTA, or CoCr particles, and viability of cells was measured by MTS 24 h after treatment (**A**). PBMCs were treated with Al_2_O_3_ particles for 24 h or 96 h (**B**). The relative cell viability was calculated as the percentage of untreated cells. The results are expressed as the mean ± SEM. *** *p* ≤ 0.001.

To evaluate how the test particles influence primary cells, we next performed experiments on human PBMCs and macrophages. First, to check whether particles affect viability of primary macrophages and human PBMCs, an MTS assay was performed 24 h post-treatment (Figure 3). Primary macrophages from two donors and human primary PBMCs from one donor were included in the study (Figure 3A,B). None of the particles affected the viability of both primary cell types at 24 h treatment (Figure 3A,B). In some experiments, the viability of primary cells even increased in the presence of particles, yet the change was not significant (Figure 3B). Interestingly, when primary PBMCs were treated for 96 h with the same concentration of particles, Al_2_O_3_ caused an increase in viability (Figure 3B). In the presence of 0.5 µm^3^/cell of Al_2_O_3_ particles the viability increased by 47% (*p* = 0.0001) in comparison with untreated cells (Figure 3B). The results indicate that Al_2_O_3_, ZTA, and CoCr nanoparticles at the concentrations used in the study (0.05–50 µm^3^/cell) are not toxic for primary human macrophages or primary human PBMCs. In fact, Al_2_O_3_ even increased PBMC viability.

### 2.4. Al_2_O_3_, ZTA, and CoCr Particles Do Not Impact TNF-α Release of Human Primary Macrophages

**Figure 4:**
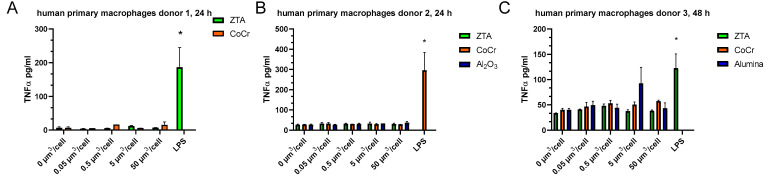
ZTA and CoCr particles do not induce TNF-α secretion in human primary macrophages. Human primary macrophages were treated with Al_2_O_3_, ZTA, or CoCr particles, and the level of TNF-α was measured in the supernatant 24 h (**A**,**B**) and 48 h (**C**) after addition of particles by ELISA. The results are expressed as the mean ± SEM. *—*p* < 0.05.

Activation of human primary macrophages by the test particles was examined by measuring secreted cytokines (TNF-α and IL-6) by ELISA and the gene expression of *IL-8* by q-PCR. Human primary macrophages from three different donors were tested. Treatment with the test particles at any test concentration (0.05, 0.5, 5, and 50 µm^3^/cell) had no significant effect on TNF-α release 24 h post-treatment of primary macrophages from donors (1 and 2) (Figure 4A,B) nor after 48 h (donor 3, Figure 4C) However, the addition of 100 ng/mL of LPS as a positive control activated TNF-α release from the same cells.

**Figure 5:**
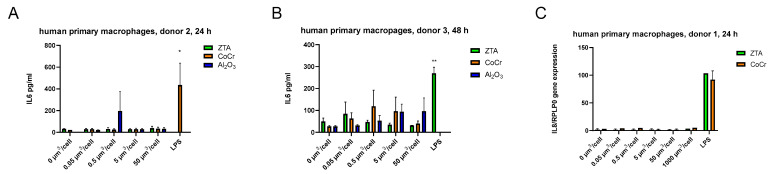
ZTA and CoCr particles do not induce IL-6 secretion or *IL-8* gene expression in human primary macrophages. Human primary macrophages were treated with CoCr, ZTA, or Al_2_O_3_ particles, and the level of IL-6 was measured in the supernatant 24 h (**A**) and 48 h (**B**) after addition of particles by ELISA. The *IL-8* gene expression level was measured by q-PCR 24 h after treatment (**C**). The results are expressed as the mean ± SEM. *—*p* < 0.05; ** *p* ≤ 0.01.

No significant particle-induced increase in IL-6 of primary macrophages from two donors was detected by ELISA (Figure 5A,B). Next, human primary macrophages were treated with CoCr and ZTA particles at concentrations of 0.05, 0.5, 5, and 50 µm^3^/cell, and the level of *IL-8* was measured 24 h after treatment. Neither CoCr nor ZTA induced *IL-8* gene expression in human primary macrophages from one donor (Figure 5C). Of note, the addition of the positive control LPS activated the release of *IL-8* from the same cells.

## 3. Discussion

The discrepancies between reports on toxicity and immunogenicity of Al_2_O_3_, ZTA, and CoCr particles led us to this study in which we compared toxicity and immunogenicity of well-defined nanoparticles from Al_2_O_3_, ZTA, and CoCr, a human THP-1 macrophage cell line, human PBMCs, and therefrom-derived primary macrophages.

Model particles in the nanometer range, mimicking the fine wear particles found in the clinical situation, were created from three different orthopaedic materials, which are commonly used in orthopaedics or dentistry. To elucidate the effect of Al_2_O_3_, ZTA, and CoCr particles on THP-1 macrophages, cell viability was assessed. Importantly, we showed that cell viability was not compromised by the test particles. Further, we assessed the cytokine expression level (*IL-8*) and measured secreted cytokines (TNF-α, IL-6) in response to the test particles by q-PCR and ELISA, respectively. Whereas ZTA particles did not cause elevated cytokine levels, CoCr caused an increase in TNF-α 48 h after treatment of THP-1 macrophages. Further, we showed that Al_2_O_3_ particles resulted in TNF-α secretion of THP-1 macrophages but not of human primary macrophages. These results are corroborated with previously published results that Al_2_O_3_ particles at high concentrations (500 µm^3^/cell) cause upregulation of TNF-α in PBMCs [31].

We measured IL-6 secretion and *IL-8* expression as additional pro-inflammatory markers but did not detect particle-induced Il-6 or IL-8 levels in human macrophages. These results differ from Jamieson et al. in which where 24 h treatment with Al_2_O_3_ nanoparticles caused upregulation of *IL-8* gene expression in THP-1 macrophages, which decreased when treated with a TLR4 inhibitor [23]. The differences could originate from several reasons. Firstly, the nanoparticles size distribution differs. Here, we employed Al_2_O_3_ nanoparticles produced in by cryo-pulverization and with an average size of 750 nm. On the other hand, the commercially available Al_2_O_3_ particles used in most of the published studies have a particle size range of 503 ± 19 nm. Secondly, in some of the experiments, LPS-activated THP-1 macrophages were employed [23] as opposed to our study in which the impact of nanoparticles were investigated solely without previous LPS activation. Thirdly, the THP-1 response to particles may differ to the response of human primary cells.

In the herein presented study, we used two different cell types, a monocytic cell line (THP-1) and primary macrophages derived from human PBMCs. Monocytic cell lines, such as THP-1, are commonly used as the model for macrophage function in immunogenicity and toxicity studies. The advantage of a monocytic cell line is ease of acquisition, expansion, and culturing. Namely, primary macrophages cannot be expanded ex vivo and are usually prepared from PBMCs isolated from large amounts of human blood. Indeed, THP-1 macrophages show similar behavior to primary macrophages derived from monocytes regarding phagocytosis and cytokine induction [32]. When the THP-1 monocytic cell line is differentiated with phorbol myristate acetate (PMA), this causes upregulation of protein kinase C (PKC) and consequently causes upregulation of genes typical for macrophages [33]. Nevertheless, we demonstrate here that THP-1 macrophages are more sensitive to Al_2_O_3_, ZTA, and CoCr nanoparticles than primary macrophages prepared from PBMCs. This aligns with previous reports that THP-1 macrophages are more susceptible to cytokine activation than primary macrophages [32]. The authors showed that the level of TNF-α was significantly higher in THP-1 macrophages than primary macrophages treated with 10 ng/mL of LPS. They proposed that THP-1 macrophages could undergo different activation stages (also alternative macrophages M2 activation besides M1 macrophages activation) than primary monocytes-derived macrophages and that direction of activation differs depending on the environment of cells. Yet, when THP-1 macrophages and primary macrophages were exposed to inactivated bacteria their responses were very similar [32]. Our results corroborate with their findings and indicate that THP-1 macrophages also undergo alternative activation in comparison with the activation of primary monocytes. Since, in vivo, several macrophages could be activated by wear debris and not only M1, we suggest that for the evaluation of immunogenicity and toxicity of nanoparticles, THP-1 macrophages represent a good model for testing ceramic nanoparticles.

An important finding of our study is that the immune response of THP-1 macrophages and primary macrophages to nanoparticles differ. Our results indicate that Al_2_O_3_, ZTA, and CoCr nanoparticles are not toxic for primary human macrophages or primary human PBMCs. Previous studies have demonstrated that ZTA and Al_2_O_3_ particles show no toxicity for PBMCs or osteoblasts [26]. Even though CoCr particles and Al_2_O_3_ particles caused elevated levels of *IL8* in THP-1 macrophages, no significant changes were observed in human primary macrophages, which suggests that THP-1 macrophages are more responsive to nanoparticles and serve as a good model for toxicity of particles.

The first group that assessed ceramic particles generated by a hip simulator was a research group from the University of Leeds [31,34]. They were also the first to compare the biological response of commercial Al_2_O_3_ particles and ZTA wear particles. Wear particles were generated in the Leeds MkII anatomical hip simulator under microseparation conditions and had bimodal sizes of 0.3–8 µm and 5–20 nm. In that study, concentrations range of particles from 0.05 to 500 µm^3^/cell were used in in vitro assays and only clinically relevant CoCr particles at 50 µm^3^/cell reduced the U937 monocyte cell viability, while other tested particles did not affect the viability [34]. An elevated TNF-α level in PBMCs, which indicates inflammation, was detected when stimulated with 100 µm^3^/cell of Al_2_O_3_ powder or 100 µm^3^/cell of wear particles. This difference was suspected to be because of fewer particles in the critical size range (0.1–1 µm) for the wear particles [31].

Of note, here we used model nanoparticles generated from the materials by cryo-pulverization. However, to better mimic the clinical situation, wear particles emerging from clinically relevant bearing situations of model hip implants under comparable load should be used as testing materials in future studies on toxicity and immunogenicity.

With the aging of the population and higher levels of age-related diseases in the world population, the number of THAs will increase in the following decades. Moreover, due to prolonged life span and THAs at younger ages (in the case of obesity), the requirement for high endurance, biocompatibility, and low toxicity of prosthetic materials for longer periods is necessary [35,36,37]. Late developed chronic PJI and loosening, either associated with particle-induced osteolysis or not, are the two most important failure modes in which periprosthetic tissue immune “health” may play an important role. Further studies are needed to decipher biochemical mechanisms of late activation of “low-grade” PJI to be able to diagnose and treat patients correctly already at the early stage of pre-clinical infection and to investigate the importance of particle-induced derangements on tissue tolerance for an implant, bone-remodelling capacity level, and bone osteolysis induction that trigger long-term implant failure. With an increase of absolute THA numbers and consequent failures, standardized protocols for evaluation of immunogenicity and toxicity of wear particles are needed. Therefore, we propose, that future efforts should be focused on the standardization of protocols for quality control assessment of prosthetic materials and modifications in the regulation.

## 4. Materials and Methods

### 4.1. Ceramic and Metallic Test Particles

**Figure 6:**
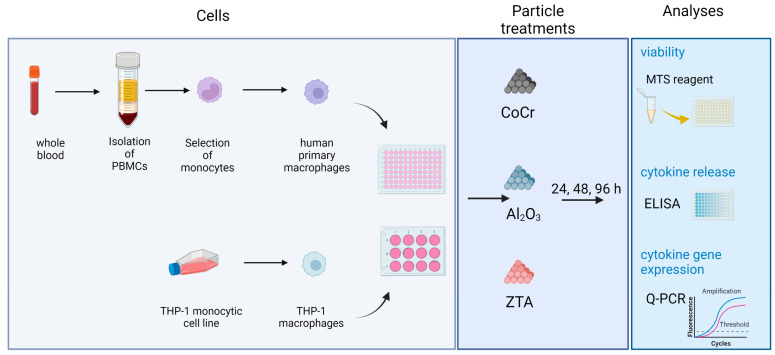
Schematic representation of the experimental design of this study. The figure was created with Biorender.com.

The Al_2_O_3_ powder consisted of alpha alumina particles, which are commercially available under the trade name CERALOX APA 05 (Condea, Hamburg, Germany). The particle size was between 150 and 550 nm with a mean size of 350 nm. A stock solution of 50 µm^3^/cell (29.5 mg of Al_2_O_3_ in 100 mL of Milli-Q water) was prepared and autoclaved at 121 °C. The solution was diluted to the other test concentrations of 5, 0.5, and 0.05 µm^3^/cell after a 10 min long ultrasound treatment. ZTA and CoCr particles were produced by cryo-pulverization by Continuum Blue Ltd. (Cardiff, UK). According to the manufacturer, the particles were guaranteed to be endotoxin free. The morphology and size of the analyzed powders were studied using field-emission scanning electron microscope (FEG-SEM, JEOL JSM 7600 F, Jeol Inc., Tokyo, Japan) equipped with Energy Dispersive X-ray Spectrometer (EDXS, INCA Oxford 350 EDS SDD, Oxford Instruments NanoAnalysis, High Wycombe, England, UK). The particle size of ZTA and CoCr, as determined by scanning electron microscopy (SEM), were 814 nm and 758 nm, respectively (Supplementary Figure S1). ZTA (density 4.37 mg/mm^3^) particle suspension was provided at the concentration of 1 mg/mL in absolute EtOH and CoCr (density 8.3 mg/mm^3^) particle suspension at the concentration of 1 mg/mL in 70% EtOH. Particles were serial diluted, and cells were treated at the concentrations of 50, 5, 0.5, and 0.05 µm^3^/cell. Additionally, a particle concentration of 1000 µm^3^/cell was used for cell viability experiments. The experimental design of the study is graphically presented in Figure 6.

### 4.2. Cell Culturing and Differentiation of THP-1 Cell Line

Human monocytic THP-1 cells (ATCC^®^ TIB-202^™^) were cultured in RPMI 1640 supplemented with 10% FBS, 1% glutamine, 1% antimycotic/antibiotic (Gibco, Thermo Fisher Scientific, Waltham, MA, USA) at 37 °C, and 5% CO_2_ and subcultured according to the manufacturers’ instructions. THP-1 monocytes were differentiated to macrophages with 100 nM phorbol 12-myristate-13-acetate (PMA) (Sigma Aldrich, St. Louis, MO, USA) treatment for 3 days.

### 4.3. Isolation of Human Peripheral Blood Mononuclear Cells (PBMCs)

PBMCs were isolated from whole blood (or buffy coats (healthy controls) using Ficoll (Ficoll^®^ Paque Plus, GE Healthcare Bio-Sciences AB, Uppsala, Sweden). Whole blood or buffy coats were diluted with equal amount of PBS and carefully applied on Ficoll (2 volumes of diluted blood on 1 volume of Ficoll) and centrifuged for 17 min at 1800 rpm without break. After centrifugation, PBMCs phase was transferred into a fresh centrifuge tube and washed with PBS following by centrifugation for 7 min 1800 rpm with break. The concentration of PBMCs was app. 10 million/5 mL of whole blood. PBMCs were immediately subjected to isolation of monocytes. Approximately 10% of PBMCs were monocytes.

### 4.4. Isolation of Human Primary Monocytes from PBMCs

Primary human monocytes were isolated from PBMCs using the Pan Monocyte Isolation kit (MACS^®^, Miltenyi Biotech, Bergisch-Gladbach, Germany) according to the manufacturers’ instructions. Before following the procedure, cells were washed twice with PBS (10 min, 300× *g* centrifugation). After isolation of monocytes, cells were either seeded for differentiation to macrophages or frozen for later experiments. Monocytes were frozen in freezing media (5% DMSO, 20% FBS, 75% full RPMI) and kept at −80 °C or liquid nitrogen for further experiments.

### 4.5. Differentiation of Human Primary Monocytes to Macrophages

Primary monocytes were thawed and diluted in full RPMI 1640 followed by centrifugation (5 min, 300× *g*) to remove freezing media. Monocytes were re-suspended in full RPMI with 100 ng/mL of rhGM-SCF (recombinant human granulocyte macrophage colony-stimulating factor) and seeded in tissue culture plates. In the 96-well plates, 50,000 cells/well (for ELISA and MTS measurements) and in the 12-well plates, 500,000 cells/well were seeded. Monocytes were differentiated into M1 macrophages for at least 10 days. M1 macrophages were treated with particles, as described above.

### 4.6. Treatment of Cells with Metallic and Ceramic Particles

Particle’s suspension stock solutions were prepared in water and kept at 4 °C. Immediately before treatment, particle suspensions were sonicated for 10 min and added to the cells at final concentrations of 0.05, 0.5, 5, and 50 µm^3^/cell in 96-well plates. Cells were treated with particles for 24, 48, or 96 h before collection and freezing of cell supernatant for cytokine analysis. In the case of RNA analysis, cells were seeded in 12-well plates after the treatment of cells with particles; cells were harvested and subjected to RNA isolation or kept at −80 °C. THP-1 monocytes, THP-1 macrophages, or human primary macrophages were treated with particle suspensions. In all the experiments, positive control lipopolysaccharide (LPS, final concentration 100 ng/µL; some experiments also 10 ng/µL) treatment was included. In negative controls, cells were left untreated. All treatments were performed in at least three biological repeats in parallel.

### 4.7. RNA Isolation and Quantitative PCR

The RNA was extracted from cells, and the complementary DNAs (cDNAs) were synthesized using High-Capacity cDNA Reverse Transcription kits (Thermo Fisher Scientific, Waltham, MA, USA) with gene expression analyses performed, as described below. For qPCR, 5× Hot FirePol EvaGreen qPCR Mix Plus (Solis, BioDyne, Tartu, Estonia) was used following the manufacturers’ instructions on a LightCycler 480 (Roche Diagnostics, Rotkreuz, Switzerland). All the samples were quantified in triplicate. Dilution series of cDNAs were prepared to create a relative standard curve, and absolute quantification of the data was performed using the second derivative maximum method (LightCycler 480, Software version 1.5; Roche Diagnostics). All data were normalized to the internal housekeeping gene of ribosomal protein, large, P0 (RPLP0). Expressions of *IL-8* and *TNF-α* were analyzed using qPCR.

### 4.8. Enzyme-Linked Immunosorbent Assay

The level of cytokines (TNF-α and IL-6) secreted from particle-treated cells was measured with enzyme-linked immunosorbent assay (ELISA) according to the manufacturers’ instructions (Invitrogen, Waltham, MA, USA). The supernatants of the treated cells were collected and stored at −80 °C before ELISA was performed. The absorbance was measured by BIO-TEK Synergy HT (Fisher Scientific, Pittsburgh, PA, USA) at 405 and 490 nm. The concentration of cytokines was determined from the standard curve.

### 4.9. Viability Assay

Cell viability was measured by CellTiter 96^®^ AQ_ueous_ One Solution kit (Promega, Madison, WI, USA) following manufacturers’ instructions. Briefly, after treatment of cells with particles, the supernatant was aspirated from the cells, and CellTiter AQ One solution diluted full RPMI media was added and incubated for 2–3 h at 37 °C and 5% CO_2_. Absorbance was measured at 570 nm by BIO-TEK Synergy HT.

### 4.10. Statistics

Statistical analyses were performed using Prism 7.0 (GraphPad Software, La Jolla, CA, USA). Data are presented as mean ± SEM. Statistical significance was determined using Student’s t-test, one-way or two-way ANOVA, followed by Bonferroni or Tukey’s post-hoc tests. Statistical significance is displayed as follows: ns—not significant (*p* > 0.05); * *p* ≤ 0.05; ** *p* ≤ 0.01; *** *p* ≤ 0.001.

## Data Availability

The data presented in this study are available on request from the corresponding authors. The data are not publicly available due to privacy-related restrictions.

## Supplementary Materials

The supporting information can be downloaded at: https://www.mdpi.com/article/10.3390/ijms24076482/s1.
