# Supplementary figures and images for: Zirconia-Toughened Alumina Ceramic Wear Particles Do Not Elicit Inflammatory Responses in Human Macrophages
Source: Int J Mol Sci. 2023 Mar 30;24(7):6482. doi: 10.3390/ijms24076482 (PMC10095128; doi:10.3390/ijms24076482)

Supplementary Figure S1. SEM pictures of ZTA and CoCr particles.

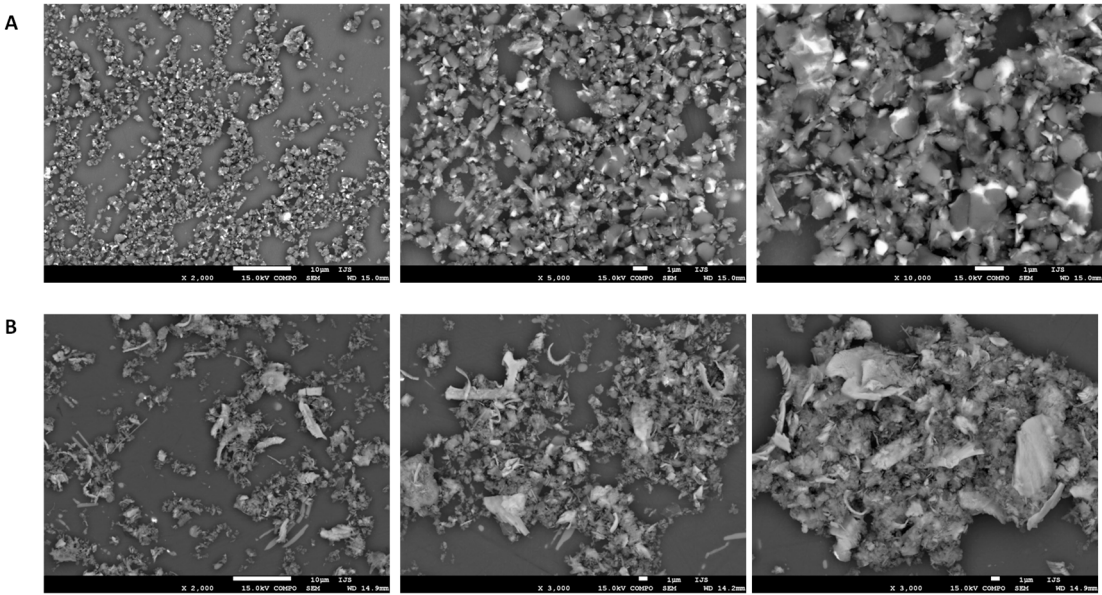

Supplement: Supplementary file 1 [file ijms-24-06482-s001.zip › ijms-2303567-supplementary.pdf]
